# Supplementary material for: Wide range screening of algorithmic bias in word embedding models using large sentiment lexicons reveals underreported bias types
Source: PLoS One. 2020 Apr 21;15(4):e0231189. doi: 10.1371/journal.pone.0231189 (PMC7173861; doi:10.1371/journal.pone.0231189)
Supplement: S1 Appendix — (DOCX) [file pone.0231189.s001.docx]

Wide range screening of algorithmic bias in word embedding models using large sentiment lexicons reveals underreported bias types

**Authors:** David Rozado^1^*

# Affiliations

^1^Otago Polytechnic, New Zealand.

*Correspondence to: [drozado@gmail.com](mailto:drozado@gmail.com)

# Supplementary material

**Content analysis of scholarly literature on the topic of algorithmic bias in word embeddings and the bias types that they cite**

Table 1 A search of the scientific literature using the queries *word embeddings bias* and *word vectors bias* revealed 28 papers addressing the topic of bias in word embeddings. The manuscripts were tabulated according to which manuscripts mentioned in their Titles, Abstracts or Introduction sections a given type of bias.

**Testing for algorithmic bias in word embeddings using the HGI lexicon - Detailed results**

This table provides specific numerical results of the association experiments summarized in Figure 3 of the main manuscript

Table 2 Spearman correlation coefficients between positive/negative labels in the HGI lexicon (N=3623) and the projection values of the HGI terms on the cultural axes (rows) representing demographic groups for 7 popular pre-trained word embedding models (columns). The column with the heading *Average* *Corr* aggregates the correlation coefficients of all embedding models. Positive correlations denote association of positive HGI terms with Pole 2 and/or association of negative HGI terms with Pole 1. Negative correlations denote association of positive HGI terms with Pole 1 and/or association of negative HGI terms with Pole 2. The complete list of words used to build the poles is provided in this Appendix.

**Correlation of cultural axes association experiments between different embedding models**

Table 3 The experimental results reported in Table 2 are highly correlated with each other across embedding models, suggesting that the different models analyzed contain similar bias types and directions despite the models having been trained on different corpora.

**Testing for algorithmic bias using an ensemble of 17 different sentiment lexicons**

Several sentiment lexicons other than HGI were projected on the cultural axes analyzed in this work. Detailed results are provided in Table 4.

Table 4 Average Spearman correlation coefficients between positive/negative labels in 17 external sentiment lexicons (columns), and the projection values of the lexicons terms on the cultural axes analyzed (rows) for 7 popular pre-trained word embedding models. The column with the heading *Average* aggregates the correlation coefficients of all lexicons in a row. Positive correlations denote association of positive lexicons terms with Pole 2 and/or association of negative terms with Pole 1. Negative correlations denote association of positive lexicons terms with Pole 1 and/or association of negative terms with Pole 2. The complete list of words used to build the poles and details about the sentiment lexicons used are provided in this Appendix.

**Correlation between association experiments using 17 sentiment lexicons**

Table 5 The experimental results reported in Table 4 are highly correlated with each other, suggesting that the different lexicons are measuring similar latent associations of negativity and positivity within embedding models.

**Building Axes to test for bias in popular word embedding models**

A comprehensive set of cultural/demographic axes have been used in this work. Some cultural axes were designed for illustration purposes (see Figure 1 and Figure 2 on the main manuscript) and others were designed specifically to detect different bias types for specific demographic groups such as those due to gender, race/ethnicity, sexual orientation, religiosity, age, socioeconomic status, physical appearance (i.e. looks), and political orientation (see Figure 3 on main manuscript). The list of axes created and the poles used to build them (as described in Figure 8 of the main manuscript) are detailed below:

Axis name: Gender – males and females

Pole 1 (male): man, men, male, males

Pole 2 (female): woman, women, female, females

Axis name: Gender - family members

Pole 1 (male family members): father, fathers, dad, dads, son, sons, brother, brothers, husband, husbands, uncle, uncles, grandfather, grandfathers, grandson, grandsons, nephew, nephews

Pole 2 (female family members): mother, mothers, mom, moms, daughter, daughters, sister, sisters, wife, wives, aunt, aunts, grandmother, grandmothers, granddaughter, granddaughters, niece, nieces

Axis name: Gender - young age

Pole 1 (young males): boy, boys

Pole 2 (young females): girl, girls

Axis name: Gender - masculinity and femininity

Pole 1 (masculinity): masculine, masculinity

Pole 2 (femininity): feminine, femininity

Axis name: Gender - popular male and female given names

Note: 200 most popular masculine and feminine names of children born between 1960-1969

Shackleford, Michael W., A.S.A., Name Distributions in the Social Security Area (1998). <https://www.galbithink.org/names/us200.htm>

Pole 1 (masculine names): Michael, John, James, Robert, David, William, Mark, Richard, Jeffrey, Stephen, Joseph, Thomas, Daniel, Timothy, Brian, Christop, Scott, Charles, Paul, Kenneth, Ronald, Anthony, Donald, Gregory, Edward, Gary, Laurence, Eric, Douglas, Patrick, Terence, Todd, Matthew, George, Keith, Andrew, Allan, Frank, Raymond, Shawn, Dennis, Daryl, Philip, Jerry, Peter, Lewis, Carl, Craig, Roger, Bruce, Tony, Glen, Rodney, Daren, Steve, Russell, Troy, Samuel, Harry, Gerald, Wayne, Leonard, Dale, Randall, Duane, Martin, Vincent, Bradley, Curtis, Walter, Barry, Jason, Dean, Victor, Jay, Juan, Derek, Carlos, Theodore, Roy, Henry, Arthur, Benjamin, Jack, Greg, Albert, Francis, Joel, Ralph, Ernest, Eugene, Stanley, Marvin, Howard, Edwin, Alexande, Brent, Kurt, Aaron, Nathan, Anton, Nicholas, Melvin, Reginald, Brett, Rick, Mitchell, Norman, Neil, Adam, Calvin, Jerome, Kirk, Brad, Clifford, Manuel, Hector, Earl, Alfred, Gilbert, Stewart, Lance, Wesley, Miguel, Kent, Warren, Andre, Clarence, Tyrone, Reuben, Bernard, Kyle, Kerry, Chad, Jorge, Alvin, Leroy, Gordon, Shane, Erik, Pedro, Jesus, Gene, Dave, Guy, Maurice, Mario, Lonnie, Leslie, Herbert, Lloyd, Vernon, Perry, Rafael, Ramon, Rickey, Wade, Dwight, Gregg, Ron, Marty, Travis, Loren, Joey, Ken, Timmy, Nelson, Kelvin, Byron, Doug, Randal, Oscar, Donnie, Ryan, Hugh, Raul, Floyd, Damien, Milton, Lester, Clinton, Orlando, Arnold, Jimmie, Jackie, Felix, Corey, Gerard, Roderick, Javier, Roland, Clyde, Ross, Jody, Clayton, Ferdinan, Herman, Nick, Julio, Wendell

Pole 2 (feminine names): Elizabet, Mary, Catherin, Deborah, Susan, Christin, Ann, Jane, Karen, Patricia, Cynthia, Laura, Theresa, Lori, Linda, Tami, Caroline, Sandra, Angel, Julia, Donna, Sherry, Pamela, Jennifer, Brenda, Cheryl, Barbara, Sharon, Margaret, Nancy, Joan, Rebecca, Diane, Victoria, Denise, Tina, Amy, Jacqueli, Milicent, Teri, Dawn, Ellen, Rose, Rhonda, Paula, Stephani, Wendy, Dinah, Sheila, Judy, Lyn, Staci, Alice, Constanc, Keri, Sarah, Jill, Karla, Dara, Joyce, Georgina, Janice, Valerie, Shelly, Martha, Wanda, Monica, Bonnie, Regina, Jodi, Sonia, Betty, Gail, Beverly, Evelyn, Colleen, Gloria, Penelope, Frances, Lou, Roberta, Shirley, Tamar, Ruth, Nora, Maureen, Marci, Helen, Veronica, Melanie, Dorothy, Virginia, Melinda, Tonya, Gwen, Sylvia, Rachel, Holly, Heather, Yolanda, Heidi, Rita, Toni, Yvonne, Jamie, Marilyn, April, Crystal, Sally, Yvette, Tania, Phyllis, Charlott, Nicole, Charlene, Belinda, Janine, Audrey, Bridget, Marla, Lily, Shawna, Vanessa, Candice, Lucy, Juana, Emily, Doris, Glenda, Casandra, Felicia, Clara, Natalia, Melody, Amanda, Wilma, Grace, Cecilia, Irene, Rox, Kay, Vivian, Geri, Arlene, Doreen, Ramona, Leta, Tara, Lydia, Josephin, Lois, Danielle, Lauren, Ada, Sabina, Katrina, Berna, Esther, Chris, Dolores, Claud, Adriana, Delores, Edith, Erica, Samantha, June, Ginger, Iris, Mildred, Priscill, Ruby, Trina, Nina, Geraldin, Rochelle, Naomi, Gretchen, Beatrice, Irma, Hope, Lana, Edna, Dahlia, Tiffany, Mona, Maribel, Florence, Olga, Alisa, Camilla, Faith, Cora, Dora, Lourdes, Myra, Alma, Nadine, Celeste, Desiree

Axis name: Race/ethnicity - Whites and African Americans

Pole 1 (Whites): White, Whites, White_American, White_Americans, white, whites, white_american, white_americans

Pole 2 (Blacks): Black, Blacks, African_American, African_Americans, Black_American, Black_Americans, black, blacks, african_american, african_americans, black_american, black_americans

Axis name: Race/ethnicity - popular given names among African-Americans and Whites

Note: list of names from reference *(8)* in main manuscript

Pole 1 (European names): Adam, Chip, Harry, Josh, Roger, Alan, Frank, Ian, Justin, Ryan, Andrew, Fred, Jack, Matthew, Stephen, Brad, Greg, Jed, Paul, Todd, Brandon, Hank, Jonathan, Peter, Wilbur, Amanda, Courtney, Heather, Melanie, Sara, Amber, Crystal, Katie, Meredith, Shannon, Betsy, Donna, Kristin, Nancy, Stephanie, Bobbie_Sue, Ellen, Lauren, Peggy, Sue_Ellen, Colleen, Emily, Megan, Rachel, Wendy

Pole 2 (African-American names): Alonzo, Jamel, Lerone, Percell, Theo, Alphonse, Jerome, Leroy, Rasaan, Torrance, Darnell, Lamar, Lionel, Rashaun, Tyree, Deion, Lamont, Malik, Terrence, Tyrone, Everol, Lavon, Marcellus, Terryl, Wardell, Aiesha, Lashelle, Nichelle, Shereen, Temeka, Ebony, Latisha, Shaniqua, Tameisha, Teretha, Jasmine, Latonya, Shanise, Tanisha, Tia, Lakisha, Latoya, Sharise, Tashika, Yolanda, Lashandra, Malika, Shavonn, Tawanda, Yvette

Axis name: Race/ethnicity - Whites and Hispanics

Pole 1 (Whites): White, Whites, White_American, White_Americans, white, whites, white_american, white_americans

Pole 2 (Hispanics): Hispanic, Hispanics, Latino, Latinos, Hispanic_American, Hispanic_Americans, hispanic, hispanics, latino, latinos, hispanic_american, hispanic_americans

Axis name: Race/ethnicity - Whites and Asians

Pole 1 (Whites): White, Whites, White_American, White_Americans, white, whites, white_american, white_americans

Pole 2 (Asians): Asian, Asians, Asian_American, Asian_Americans, asian, asians, asian_american, asian_americans

Axis name: Sexual orientation - heterosexuality and homosexuality

Pole 1 (heterosexuals): heterosexual, heterosexuals, heterosexuality

Pole 2 (homosexuals): homosexual, homosexuals, gay, gays, lesbian, lesbians, lgbt, lgbtq, glbt, lgb, homosexuality

Axis name: Religiosity - general

Pole 1 (religious): Christian, Christians, Christianity, Catholic, Catholics, Catholicism, Protestant, Protestants, Protestantism, Muslim, Muslims, Moslem, Moslems, Islam, Jew, Jews, Judaism, Hindu, Hindus, Hinduism, Buddhist, Buddhists, Buddhism, Mormon, Mormons, Mormonism, Evangelical, Evangelicals, Evangelicalism,

christian, christians, christianity, catholic, catholics, catholicism, protestant, protestants, protestantism, muslim, muslims, moslem, moslems, islam, jew, jews, judaism, hindu, hindus, hinduism, buddhist, buddhists, buddhism, mormon, mormons, mormonism, evangelical, evangelicals, evangelicalism

Pole 2 (nonreligious): nonreligious, secular, secularist, secularists, atheist, atheists, agnostic, agnostics, religionless, irreligious, nonbeliever, nonbelievers, non_believer

Axis name: Religiosity - Christianity and Islam

Pole 1 (christian person): Christian, Christians, Christianity, christian, christians, christianity

Pole 2 (muslim person): Muslim, Muslims, Islam, muslim, muslims, islam

Axis name: Age - old and young

Pole 1 (elder): elderly, elders, old, aged, aging, senior_citizen, senior_citizens, retired, old_age

Pole 2 (youth): youth, young, youngness, youthfulness, young_citizen, young_citizens

Axis name: Socioeconomic status

Pole 1 (working/middle class): working_class, middle_class, blue_collar, white_collar, wage_earners

Pole 2 (upper-class): upper_class, affluent, rich, wealthy, prosperous, moneyed

Axis name: Physical appearance (i.e. looks)

Pole 1 (unattractive physical appearance): unattractive, plain_looking, homely, ugly, unappealing

Pole 2 (attractive physical appearance): beautiful, handsome, cute, attractive, good_looking

Axis name: Politics - Personal ideology

Pole 1 (conservative): conservative, conservatives, right_winger, rightwinger, right_wingers, rightwingers, right_leaning, right_wing, rightwing

Pole 2 (liberal): liberal, liberals, progressive, progressives, left_winger, leftwinger, left_wingers, leftwingers, left_leaning, left_wing, leftwing

Axis name: Politics - Party affiliation

Pole 1 (Republican): Republican, Republicans, GOP, Republican_Party, Republican_voter, Republican_voters, registered_Republican, registered_Republicans, republican, republicans, gop, republican_party, republican_voter, republican_voters, registered_republican, registered_republicans

Pole 2 (Democrat): Democrat, Democrats, Democratic_Party, Democrat_voter, Democrat_voters, registered_Democrat, registered_Democrats, democrat, democrats, democratic_party, democrat_voter, democrat_voters, registered_democrat, registered_democrats

Axis name: Politics – U.S. presidents

Notes: U.S. presidents since World War II.

Pole 1 (Republicans): Dwight_Eisenhower, Eisenhower, Richard_Nixon, Nixon, Gerald_Ford, Ronald_Reagan, Reagan, George_Bush, Bush, Donald_Trump

Pole 2 (Democrats): Franklin_Roosevelt, Roosevelt, Harry_Truman, Truman, John_Kennedy, Kennedy, Lyndon_Johnson, Jimmy_Carter, Carter, Bill_Clinton, Clinton, Barack_Obama, Obama

Axis name: Politics - ideologies abstract

Pole 1 (conservatism): conservatism, neoconservatism, illiberalism, ultraconservatism, far_right

Pole 2 (liberalism): liberalism, progressivism, egalitarianism, ultraliberalism, far_left

Axis name: Politics – famous/influential liberals and conservatives

Note: list taken from the top 20 conservatives and liberals at “Top 100 US liberals and conservatives” <https://www.telegraph.co.uk/news/worldnews/northamerica/usa/6951961/Top-100-US-liberals-and-conservatives.html>

Pole 1 (famous/influential conservatives): Dick_Cheney, Cheney, Rush_Limbaugh, Limbaugh, Map_Drudge, Drudge, Sarah_Palin, Palin, Robert_Gates, Gates, Glenn_Beck, Beck, Roger_Ailes, Ailes, David_Petraeus, Petraeus, Paul_Ryan, Ryan, Tim_Pawlenty, Pawlenty, Mitt_Romney, Romney, George_Bush, Bush, John_Roberts, Roberts, Haley_Barbour, Barbour, Eric_Cantor, Cantor, John McCain, McCain, Mike_Pence, Pence, Bob_McDonnell, McDonnell, Newt_Gingrich, Gingrich, Mike_Huckabee, Huckabee

Pole 2 (famous/influential liberals): Barak_Obama, Obama, Hillary_Clinton, Clinton, Nancy_Pelosi, Pelosi, Bill_Clinton, Clinton, Rahm_Emanuel, Emanuel, Al_Gore, Gore, Oprah_Winfrey, Winfrey, Tim Geithner, Geithner, David_Axelrod, Axelrod, Harry_Reid, Reid, Michelle_Obama, Obama, Arianna_Huffington, Huffington, Sonia_Sotomayor, Sotomayor, Denis_McDonough, McDonough, Janet_Napolitano, Napolitano, Mark_Warner, Warner, Robert_Gibbs, Gibbs, Barney_Frank, Frank, John_Kerry, Kerry, Eric_Holder, Holder

**Axes used in Figures 1 and 10**

Axis name: death to life

Pole 1 (death): death, dying, decease

Pole 2 (life): alive, life, living

Axis name: poverty to economic development

Pole 1 (poverty): poor, poverty, underdeveloped

Pole 2 (economic development): wealth, rich, wealthy, prosperous, developed

Axis name: budget to expensive cars

Pole 1 (budget): affordable, budget, cheap, low_cost, poor, bargain, economical, inexpensive

Pole 2 (expensive): expensive, rich, prosperous, wealthy, affluent, luxurious, wealth, lavish, upscale, pricey

Axis name: disease to health

Pole 1 (disease): disease, sick, sickness, illness

Pole 2 (health): health, healthy, well_being

Axis name: dictatorship to democracy

Pole 1 (dictatorship): dictatorship, dictator, dictators

Pole 2 (democracy): democracy, democratic_leader, democratic_leaders, representative_government

Axis name: respectable to malevolent historical figures

Pole 1 (malevolent historical figures): Hitler, Stalin, Bin_Laden, Pol_Pot, Heinrich_Himmler, Saddam_Hussein, Joseph_Goebbels

Pole 2 (respected historical figures): Gandhi, MLK, Nelson_Mandela, Mother_Teresa, Abraham_Lincoln

**External Lexicons used**

Table 6 contains the list of sentiment lexicons used in this work. The lexicons contain terms externally annotated for positive and negative polarity. Lexicons were preprocessed to remove invalid entries such as for instance emoticons in the Vader lexicon since they are not present in the word embeddings models analyzed. All lexicons were lowercase. Preprocessing occasionally resulted in lexicon sizes slightly smaller than the original lexicons size. In the case of HGI, the smaller lexicon size is due to entries in HGI having multiple annotations for different senses. In those cases, this work used the annotation corresponding to the most frequent sense of the word. Hyphenated compound words (such as anti-social for instance) were represented with the underscore character (anti_social) and reverted back to hyphenated form when used in a word embedding model using hyphenated representations for compound words.

Table 6 External lexicons used to test for bias in popular word embeddings models.

**Alignment of Wordnet antonym pairs with cultural axes**

Figure 1 Orientation similarity between the personal ideology cultural axis and axes created from 3872 WordNet antonym pairs across seven popular word embedding models (columns). Each antonym pair axis is displayed in the Table in the form word1-word2 (cosine similarity). The top 30 antonym pairs are ordered in terms of their axis cosine similarity to the personal ideology cultural axis for each embedding model. A high rank antonym pair axis reflects a preferential association in the embedding model of word1 in the antonym pair with Pole1 (representing conservatives) of the cultural axis and word2 with Pole2 (representing liberals).

Figure 2 Orientation similarity between the male to female cultural axis and axes created from 3872 WordNet antonym pairs across seven popular word embedding models (columns). Each antonym pair axis is displayed in the Figure in the form word1-word2 (cosine similarity). The top 30 antonym pairs are ordered in terms of their axis cosine similarity to the male to female cultural axis for each embedding model. A high rank antonym pair axis reflects a preferential association in the embedding model of word1 in the antonym pair with Pole1 (representing males) of the cultural axis and word2 with Pole2 (representing females).

Figure 3 Orientation similarity between the popular given names among Whites to popular given names among African-Americans cultural axis and axes created from 3872 WordNet antonym pairs across seven popular word embedding models (columns). Each antonym pair axis is displayed in the Figure in the form word1-word2 (cosine similarity). The top 30 antonym pairs are ordered in terms of their axis cosine similarity to the cultural axis for each embedding model. A high rank antonym pair axis reflects a preferential association in the embedding model of word1 in the antonym pair with Pole1 (representing popular given names among Whites) of the cultural axis and word2 with Pole2 (representing popular given names among African-Americans).

Figure 4 Orientation similarity between the religiosity axis and axes created from 3872 WordNet antonym pairs across seven popular word embedding models (columns). Each antonym pair axis is displayed in the Figure in the form word1-word2 (cosine similarity). The top 30 antonym pairs are ordered in terms of their axis cosine similarity to the religiosity cultural axis for each embedding model. A high rank antonym pair axis reflects a preferential association in the embedding model of word1 in the antonym pair with Pole1 (representing religious sentiment) of the cultural axis and word2 with Pole2 (representing lack of religious sentiment).

Figure 5 Orientation similarity between the Christians to Muslims cultural axis and axes created from 3872 WordNet antonym pairs across seven popular word embedding models (columns). Each antonym pair axis is displayed in the Figure in the form word1-word2 (cosine similarity). The top 30 antonym pairs are ordered in terms of their axis cosine similarity to the Christians to Muslims cultural axis for each embedding model. A high rank antonym pair axis reflects a preferential association in the embedding model of word1 in the antonym pair with Pole1 (representing Christians) of the cultural axis and word2 with Pole2 (representing Muslims).

Figure 6 Orientation similarity between the socioeconomic status cultural axis and axes created from 3872 WordNet antonym pairs across seven popular word embedding models (columns). Each antonym pair axis is displayed in the Figure in the form word1-word2 (cosine similarity). The top 30 antonym pairs are ordered in terms of their axis cosine similarity to the socioeconomic status cultural axis for each embedding model. A high rank antonym pair axis reflects a preferential association in the embedding model of word1 in the antonym pair with Pole1 (representing working and middle-class) of the cultural axis and word2 with Pole2 (representing upper-class).

**Projection of WordNet antonym pairs within the HGI lexicon that better align with cultural axes representing demographic groups**





Figure 7 HGI sentiment lexicon words among Wordnet top 100 antonym pairs derived axes that better align in orientation with the males to females cultural axis in 7 popular pre-trained embedding models. Words have been color-coded red and blue to signify negative and positive labels.





Figure 8 HGI sentiment lexicon words among Wordnet top 100 antonym pairs derived axes that better align in orientation with the masculinity to femininity cultural axis in 7 popular pre-trained embedding models. Words have been color-coded red and blue to signify negative and positive labels.





Figure 9 HGI sentiment lexicon words among Wordnet top 100 antonym pairs derived axes that better align in orientation with the Whites to African-Americans cultural axis in 7 popular pre-trained embedding models. Words have been color-coded red and blue to signify negative and positive labels.





Figure 10 HGI sentiment lexicon words among Wordnet top 100 antonym pairs derived axes that better align in orientation with the popular given names among Whites and African-Americans cultural axis in 7 popular pre-trained embedding models. Words have been color-coded red and blue to signify negative and positive labels.





Figure 11 HGI sentiment lexicon words among Wordnet top 100 antonym pairs derived axes that better align in orientation with the religiosity cultural axis in 7 popular pre-trained embedding models. Words have been color-coded red and blue to signify negative and positive labels.





Figure 12 HGI sentiment lexicon words among Wordnet top 100 antonym pairs derived axes that better align in orientation with the Christians to Muslims cultural axis in 7 popular pre-trained embedding models. Words have been color-coded red and blue to signify negative and positive labels.





Figure 13 HGI sentiment lexicon words among Wordnet top 100 antonym pairs derived axes that better align in orientation with the age cultural axis in 7 popular pre-trained embedding models. Words have been color-coded red and blue to signify negative and positive labels.





Figure 14 HGI sentiment lexicon words among Wordnet top 100 antonym pairs derived axes that better align in orientation with the physical appearance cultural axis in 7 popular pre-trained embedding models. Words have been color-coded red and blue to signify negative and positive labels.





Figure 15 HGI sentiment lexicon words among Wordnet top 100 antonym pairs derived axes that better align in orientation with the socioeconomic status cultural axis in 7 popular pre-trained embedding models. Words have been color-coded red and blue to signify negative and positive labels. The words ‘rich’ and ‘cut’ have been left out from the plot since they were outliers in several axes that distorted the visualization.
